# A novel microtubule de-stabilizing complementarity-determining region C36L1 peptide displays antitumor activity against melanoma *in vitro* and *in vivo*

**DOI:** 10.1038/srep14310

**Published:** 2015-09-22

**Authors:** Carlos R. Figueiredo, Alisson L. Matsuo, Ricardo A. Azevedo, Mariana H. Massaoka, Natalia Girola, Luciano Polonelli, Luiz R. Travassos

**Affiliations:** 1 Department of Microbiology, Immunology and Parasitology, Federal University of São Paulo (UNIFESP), São Paulo, São Paulo, Brazil; 2 Department of Immunology, Institute of Biomedical Sciences, University of São Paulo, São Paulo, SP, Brazil; 3 Recepta Biopharma, São Paulo, SP, Brazil; 4 Microbiology and Virology Unit, Department of Biomedical, Biotechnological and Translational Sciences, Universitá degli Studi di Parma, Parma, Italy

## Abstract

Short peptide sequences from complementarity-determining regions (CDRs) of different immunoglobulins may exert anti-infective, immunomodulatory and antitumor activities regardless of the specificity of the original monoclonal antibody (mAb). In this sense, they resemble early molecules of innate immunity. C36L1 was identified as a bioactive light-chain CDR1 peptide by screening 19 conserved CDR sequences targeting murine B16F10-Nex2 melanoma. The 17-amino acid peptide is readily taken up by melanoma cells and acts on microtubules causing depolymerization, stress of the endoplasmic reticulum and intrinsic apoptosis. At low concentrations, C36L1 inhibited migration, invasion and proliferation of B16F10-Nex2 cells with cell cycle arrest at G2/M phase, by regulating the PI3K/Akt signaling axis involving Rho-GTPase and PTEN mediation. Peritumor injection of the peptide delayed growth of subcutaneously grafted melanoma cells. Intraperitoneal administration of C36L1 induced a significant immune-response dependent anti-tumor protection in a syngeneic metastatic melanoma model. Dendritic cells stimulated *ex-vivo* by the peptide and transferred to animals challenged with tumor cells were equally effective. The C36 V_L_ CDR1 peptide is a promising microtubule-interacting drug that induces tumor cell death by apoptosis and inhibits metastases of highly aggressive melanoma cells.

Short peptide sequences of the complementarity determining regions of immunoglobulins (CDRs) have been described to display antimicrobial, antiviral and antitumor activities, independently of the specificity of the original antibody^1^. These molecules, therefore, are expected to be natural, unlimited sources of peptides potentially active against infectious agents and tumor cells^2^,^3^. Peptides and small molecules may have advantages over monoclonal antibodies on the ability to penetrate solid cancers^4^, in addition to their easy synthesis in a purified grade, versatility of chemical modification, tumor-penetrating ability and good compatibility^5^. They are increasingly focused on as a platform of drugs for treatment of diabetes, cardiovascular diseases and cancer. Peptides may act on tumor cells in many different ways^5^,^6^, by exerting direct cytotoxicity attributed to induced restriction of tumor growth, inhibition of angiogenesis, cell damage caused by interactions with proteins, enzymes, signal transduction mediators and the gene expression machinery^7^,^8^,^9^. Moreover, peptides have been shown to act as anti-infective agents in mouse models or inhibit growth of tumors, inducing cytotoxicity by different mechanisms, including programmed cell death (apoptosis)^10^.

Frequent targets of antitumor peptides are the constituents of the cytoskeleton, such as actin and microtubules (MTs). Currently used anti-cancer drugs targeting the cytoskeleton, may either stabilize or de-stabilize MTs thus inhibiting cell proliferation and inducing cell death^11^. We have recently characterized an antitumor peptide (C7H2) that binds to β-actin and interferes in actin dynamics thus leading to cell apoptosis^12^. This peptide is a V_H_ CDR 2 from mAb C7, raised against *Candida albicans* antigens^1^,^3^. It exerted anti-tumor activities *in vitro* and *in vivo* agains*t* murine B16F10-Nex2 melanoma and was cytotoxic to human cancer cell lineages.

Current clinical data attesting the efficiency of peptide-based cancer vaccines have increased, in the last decade^13^. Peptides have been used as direct cytotoxic or tumor-targeting agents, angiogenesis inhibitors, carriers of drugs and radionuclides, agents acting on tumor hormonal response and anticancer immune therapy. Peptides based on immunoglobulin CDRs and other internal Ig sequences represent a rich source of bioactive molecules that may exert antitumor activities and immunomodulatory effects *in vitro* and *in vivo*^1^,^14^,^15^,^16^,^17^,^18^.

When acting directly on tumor cells, mostly melanoma, peptides derived from immunoglobulins and from transcription factors may display cytotoxic effects including growth arrest, inhibition of migration and invasion, induction of apoptosis/necrosis and cell senescence 1,3,12,14,15,16.

In the present work, we investigated the mechanism of action of the antitumor V_L_ CDR 1 of an anti-vaccinia C36 IgG, recognized as a novel microtubule de-stabilizing CDR peptide. This CDR has a peptide sequence highly conserved in different immunoglobulin light kappa chains. Clone 36 (C36) was obtained from a combinatorial phage-display library of human Fab antibody fragments generated from IgG heavy- and light-chain genes cloned from the lymphocytes of a vaccinia virus immune donor^19^.

## Results

### Apoptotic effects induced by C36L1

**Figure 1:**
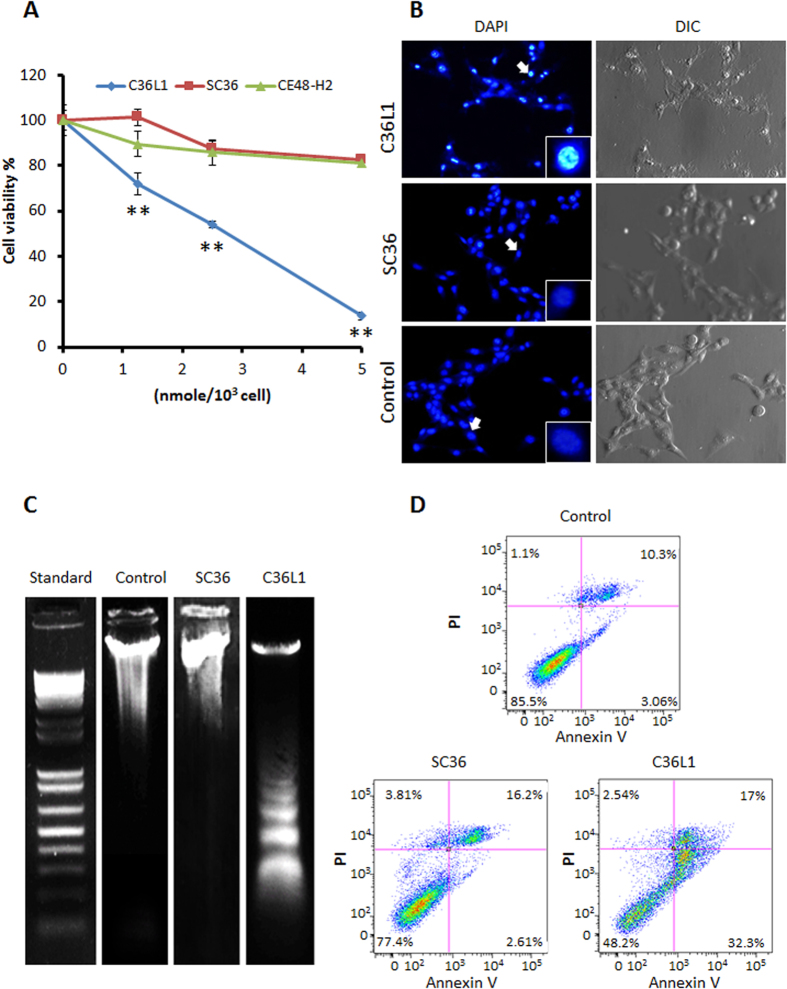
Apoptotic effects of C36L1 in B16F10-Nex2 cells. (**A**) Cytotoxicity of C36L1, SC36 and CE48-H2 peptides at different concentrations ranging from 0 to 5 nmoles/10^3^ cells. (**p < 0.01 in relation to CE48-H2 irrelevant CDR peptide control); (**B**) Chromatin condensation in B16F10-Nex2 cells treated with C36L1 at 7.5 nmoles/10^3^ cells. (**C**) DNA fragmentation in B16F10-Nex2 cells treated with C36L1 at 6 nmoles/10^3^ cells. (**D**) Translocation of phosphatidylserine in B16F10-Nex2 previously incubated with C36L1 and SC36 control peptide at 1 nmole/10^3^ cells for 18 h.

Synthetic peptide C36L1 based on the V_L_ CDR1 of Fab C36^19^, significantly reduced lung colony formation of B16F10-Nex2 melanoma cells *in vivo* and was cytotoxic to several human cancer cells *in vitro*, as well as murine melanoma^15^. In the present work, we investigated the mechanisms of antitumor activity of C36L1. We found that when B16F10-Nex2 cells are incubated with C36L1 at 2 nmoles/10^3^ cells (IC_50_), both cytotoxic and cytostatic effects are shown, as evidenced by real time microscopy in 6 h of incubation (Supplementary Video S1). We showed that the peptide conserved amino acid sequence is essential for the observed cytotoxic effects since the scrambled control peptide SC36 was inactive, as compared to an irrelevant CDR peptide CE48-H2 (Fig. 1a). Examining morphological and biochemical alterations in tumor cells, we suggested the type of cell death induced by high concentrations of C36L1 in B16F10-Nex2 melanoma cells. Chromatin condensation was observed in 40% of the tumor cells treated for 5 h with C36L1 at 7.5 nmoles/10^3^ cells (Fig. 1b). No changes were induced by the scramble SC36 peptide. DNA degradation by 6 nmoles/10^3^ cells C36L1 occurred in a typical ladder pattern (Fig. 1c). Externalized phosphatidylserine (PS), indicating early apoptosis and late membrane increased permeability were determined by annexin V binding and propidium iodide nuclear staining, respectively. A significant increase in the number of annexin V-positive cells after treatment with C36L1 at 1 nmole/10^3^ cells, as compared to SC36 and the untreated control was observed (Fig. 1d).

### Morphological and functional alterations in ER and mitochondria

**Figure 2:**
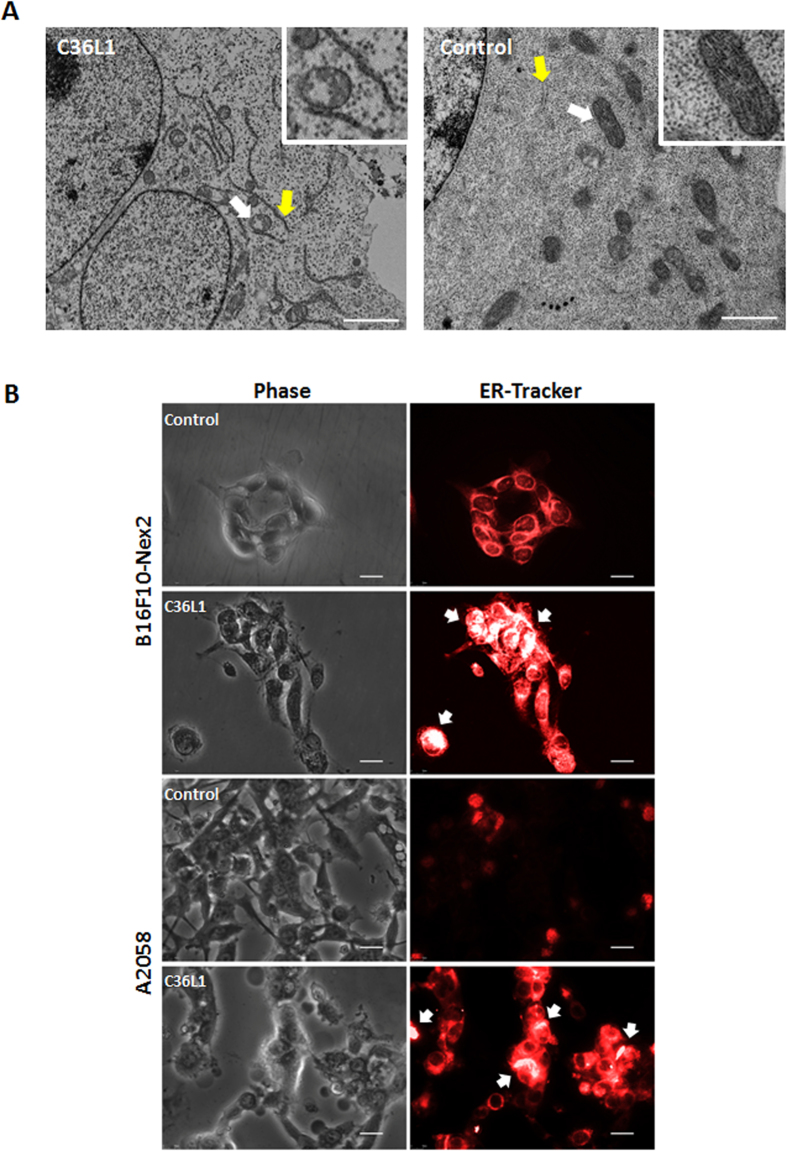
Effects on mitochondria and endoplasmic reticulum (ER). (**A**) Transmission electron microscopy (TEM) of B16F10-Nex2 previously incubated with C36L1 at 1.2 nmoles/10^3^ cells for 18 h. White arrows, normal and altered vacuolated mitochondria; yellow arrow, normal and condensed ER; Scale bar represents 2 μm; (**B**) C36L1 induces ER condensation of cells of B16F10-Nex2 and A2058 cells. Tumor cells were previously incubated with peptide C36L1 at 5 nmoles/10^3^ for 3 h and further stained with fluorescent ER-tracker. The morphology of ER was documented by fluorescence microscopy (Nikon Instruments, Inc, Melville, NY). White arrow indicates high condensation area. Scale bar represents 20 μm.

**Figure 3:**
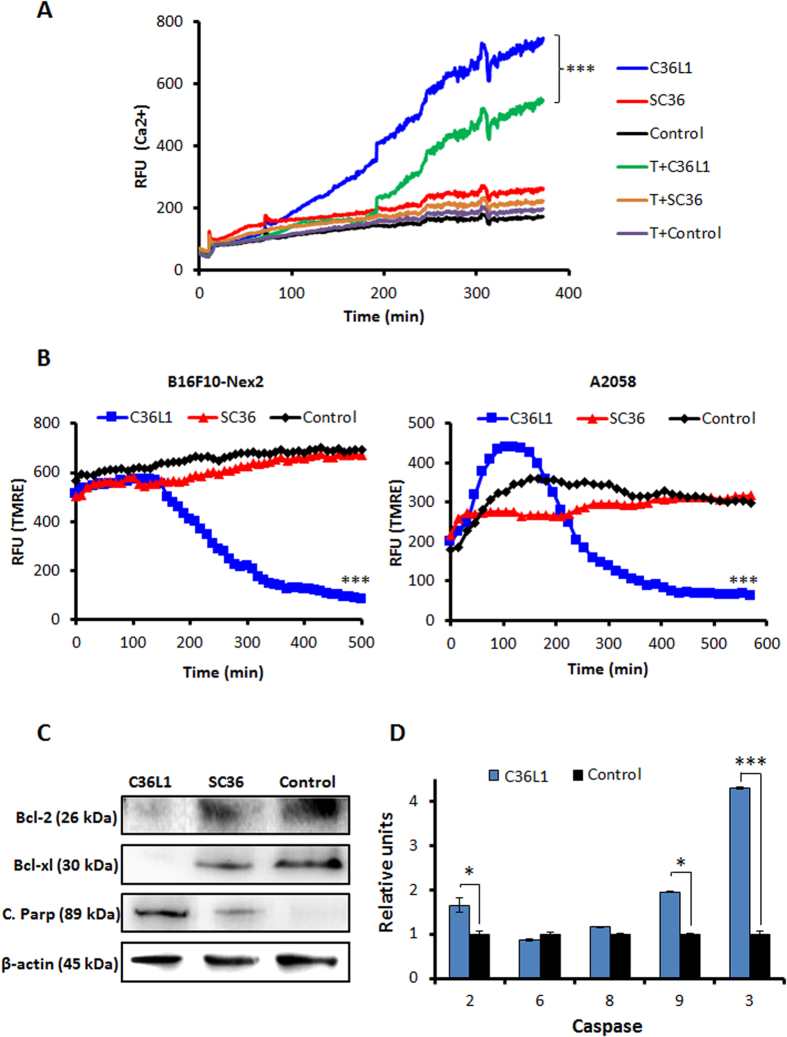
Functinoal effects of C36L1 in ER and mitochondria. (**A**) C36L1 induces ER Ca^2+^ release in B16F10-Nex2 cells, added at 5 nmoles/10^3^ cells for 6 h (***p < 0.001, in relation to T (thapsigargin) + C36L1 group); (B) C36L1 induces collapse of the mitochondrial transmembrane potential (∆ψm) in B16F10-Nex2 and A2058 cells during treatment at 5 nmoles/10^3^ cells for 6h (***p < 0.001, in relation to untreated control); (**C**) Lysates from B16F10-Nex2 cells, previously treated with peptides at 5 nmoles/10^3^ cells for 18 h at 37 °C were analyzed for prosurvival and proapoptotic proteins by Western blotting with the following antibodies: Bcl-2, Bcl-xl and C. Parp (Cleaved Parp). Anti β-actin was used as total protein loading control (***p < 0.001, in relation to untreated control). (**D**) Caspase-2, −6, −8, −9 and −3 activity in B16F10-Nex2 cells incubated with C36L1 at 1.2 nmole/10^3^cells for 2 h (*p < 0.05; **p < 0.01; **p < 0.001, in relation to untreated control).

Transmission electron microscopy (TEM) of C36L1-treated B16F10-Nex2 cells, at 1.2 nmole/10^3^ cells for 18 h, showed condensed endoplasmic reticulum (ER) coupled to vacuolated mitochondria with damaged cristae (Fig. 2a). In addition, ER integrity was assessed by fluorescence microscopy in murine and human melanoma cells after incubation with C36L1 at 5 nmoles/10^3^ cells. ER condensation is indicated in Fig. 2b by white arrows after 3 h of treatment. High cytosolic Ca^2+^ induced by depletion of ER Ca^2+^ led to mitochondrial disruption and apoptosis. Since C36L1 is apoptotic and causes structural alterations in ER membrane and mitochondria we examined the effects of the peptide on the cytosolic Ca^2+^ and mitochondrial permeability. C36L1-treated B16F10-Nex2 cells showed a significant increase in the levels of cytosolic Ca^2+^, which was released mainly from the ER, since this effect was obtained in a calcium-free buffer and was reduced after pre-incubation of cells with Thapsigargin (T) for 1 h (Fig. 3a). The collapse of the mitochondria transmembrane potential (∆ψm) was observed in early stages of incubation with C36L1, culminating in cell death after 6 h of treatment of murine and human melanoma cells (Fig. 3b). The kinetics of ∆ψm collapse induced by C36L1 in murine and human melanoma cells can be seen in the Supplementary Videos obtained by fluorescence time-lapse microscopy (Supplementary Video S2 and S3). Simultaneously, C36L1 induced downregulation of anti-apoptotic proteins Bcl-2 and Bcl-xl, and upregulation of cleaved parp, active caspase-2, caspase-9 and caspase-3, with no significant activation of caspase 8 (Fig. 3c,d).

### Antitumor effect of low concentrations of C36L1

**Figure 4:**
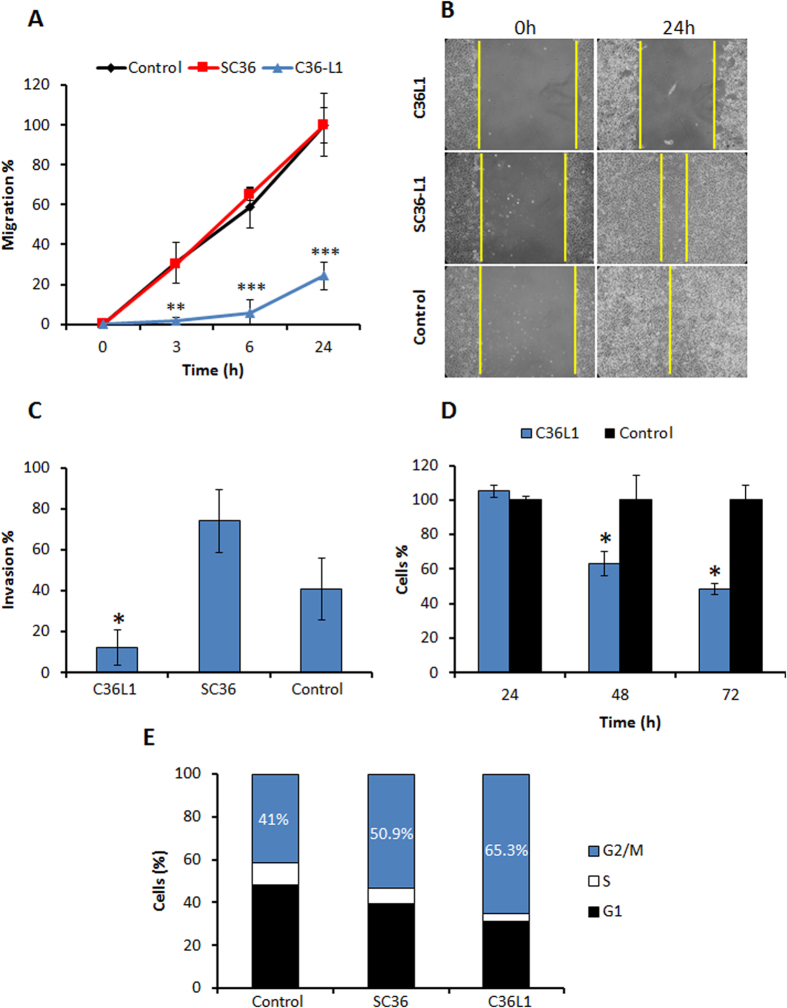
Inhibitory effects on migration, invasion and proliferation. (**A**) Migration inhibition of B16F10-Nex2 cells incubated with 0.3 nmole/10^3^ cells of C36L1 and SC36 for 24 h (**B**) Migration field examined by phase contrast microscopy at 0 and 24 h (magnification, ×100); (**C**) C36L1 inhibits invasion through Matrigel of B16F10-Nex2 cells, added 0.3 nmoles/10^3^ cells during 24 h; (**D**) Time dependent inhibition of B16F10-Nex2 cells proliferation by C36L1 at 0.5 nmoles/10^3^ cells; (**E**) Cell cycle of B16F10-Nex2 cells after incubation with C36L1 and SC36L1 at 0.5 nmoles/10^3^ cells for 18 hours. Percent tumor cells at G1, S and G2/M phases are indicated. (*p < 0.05; **p < 0.01; **p < 0.001, in relation to untreated control).

The inhibitory effect of C36L1 on tumor cell migration, a crucial step of melanoma invasion ability^20^,^21^ was examined using the wound-healing assay. A 75.7% decrease of B16F10-Nex2 cell migration was observed after 24 h incubation of tumor cells with C36L1 at 0.3 nmole/10^3^ cells, as compared to negative controls (Fig. 4a,b). Inhibition of cell invasion was then evaluated using Matrigel and also in this assay system C36L1 showed a significant activity, precluding 80% of melanoma cell invasion as compared to SC36 and the negative control (Fig. 4c). C36L1 at the low concentration of 0.5 nmole/10^3^ cells inhibited 52% of cell proliferation (Fig. 4d), with arrest of the cell cycle mainly (65.3% tumor cells) in the G2M phase after incubation with 0.5 nmole/10^3^ cells of C36L1, compared with SC36 (50.9%) and the negative control (40.1%) (Fig. 4e).

### C36L1 efficiently penetrates B16F10-Nex2 cells and reacts with tubulin

**Figure 5:**
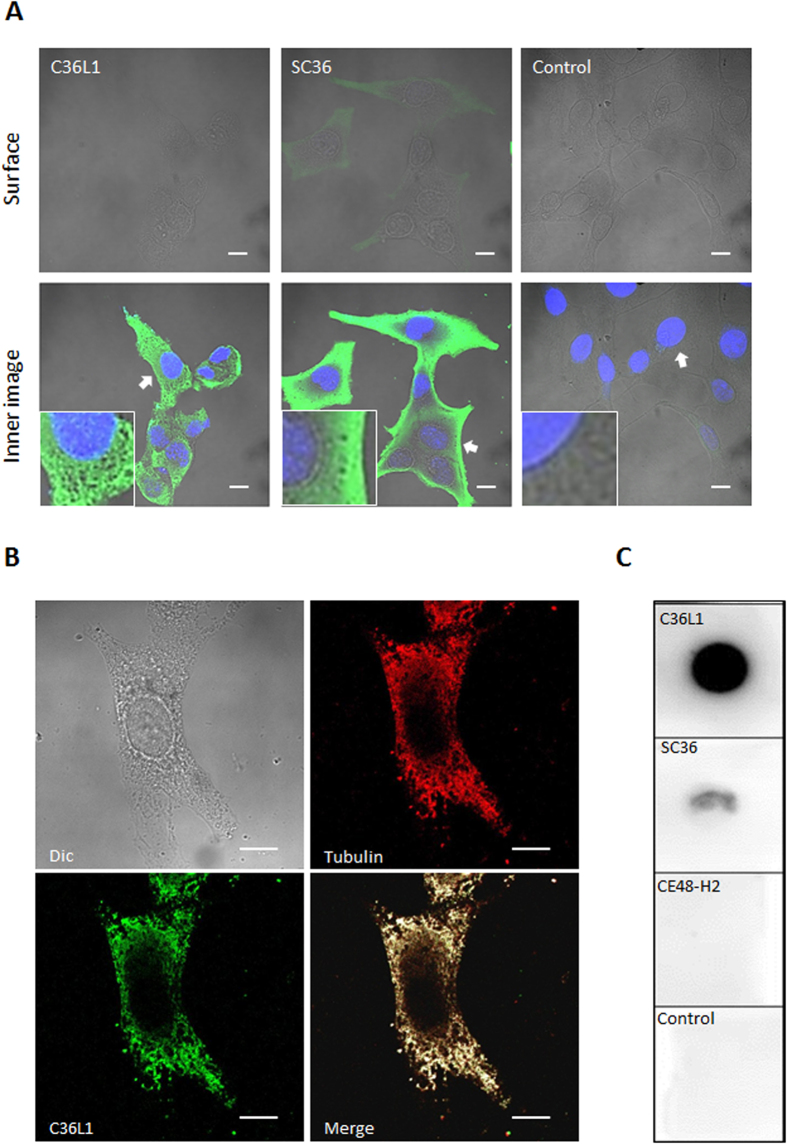
Peptide internalization and reactivity with microtubules. (**A**) C36L1 and SC36 internalization in B16F10-Nex2 cells incubated with 1.5 nmoles/10^3^ cells of biotinylated peptides for 30 min. Images were acquired by confocal fluorescence microscopy (CFM). Scale bar represents 10 μm; (**B**) C36L1 colocalizes with microtubules of B16F10-Nex2 cells analyzed by CFM (magnification, ×100). Scale bar represents 10 μm; (**C**) C36L1 binds to tubulin present in the lysate of B16F10-Nex2 cells. Dot-blots were performed by coating the nitrocellulose membranes with 3 μg of each peptide C36L1, SC36 and CFE48-H2 (inactive CDR peptide control) in 3 μL vehicle (Milli-Q water). Experimental and control dot-blots were performed as described in methods in the following order: (**a**) C36L1/tubulin; (**b**) SC36/tubulin; (**c**) CE48-H2/tubulin; (**d**) Control (vehicle). Detection was made with anti-α-tubulin mAb.

The efficacy of cell internalization of C36L1 has been determined by flow cytometry assay. 2 × 10^6^ Melanoma cells were incubated with biotinylated peptides at 0.15 nmol/10^3^ cells for 30 min and after incubation, cells were fixed in ice cold ethanol 70% and permeabilized or not in Triton X-100 0.1% in PBS. Cells were then incubated with streptavidin-FITC and analyzed by flow cytometry. We observed that 35% of permeabilized cells were positive for C36L1 compared with non-permeabilized cells (15%), showing that 20% of melanoma cells had the C36L1 peptide internalized after 30 min of incubation, compared with SC36 peptide (6% of internalized cells) (Supplementary Figure S2). We have then used confocal microscopy as a common method for detecting fluorescent targets and drugs inside cells or on their surface. Figure 5a shows surface and inner focal planes of B16F10-Nex2 cells previously incubated with a biotinylated peptide and further stained with streptavidin-FITC. We did not observe a significantly positive peptide stain on the surface focal plane as compared to the inner focal plane, which means that both peptides entered the cells within 30 min of incubation. This method allowed us to discuss the differences regarding the pattern of cytosolic distribution for both peptides in melanoma cells. The intracellular distribution pattern of C36L1 differed from that of SC36, in that it bound to a branched network of filamentous structures instead of being homogeneously spread inside the tumor cell (Fig. 5a). Such cytosolic distribution of C36L1 together with its inhibitory properties, including mitotic arrest similar to microtubule-targeted drugs^22^,^23^, suggested that the peptide interacted with the microtubule cytoskeleton. Interaction with microtubules was examined by confocal microscopy and we observed that C36L1 significantly co-localized with tubulin at the focal plane of microtubules, as compared to the SC36 scramble peptide and negative control (Fig. 5b). Interaction was further confirmed using a chemiluminescence dot blot assay. We observed that C36L1 significantly bound to microtubule structures present on B16F10-Nex2 cell lysate, compared to negative controls, the SC36 scramble peptide and an irrelevant CDR peptide (CE48-H2) (Fig. 5c), which are inactive on melanoma cells^15^.

### C36L1 disrupts microtubule cytoskeleton and inhibits recombinant tubulin polymerization

**Figure 6:**
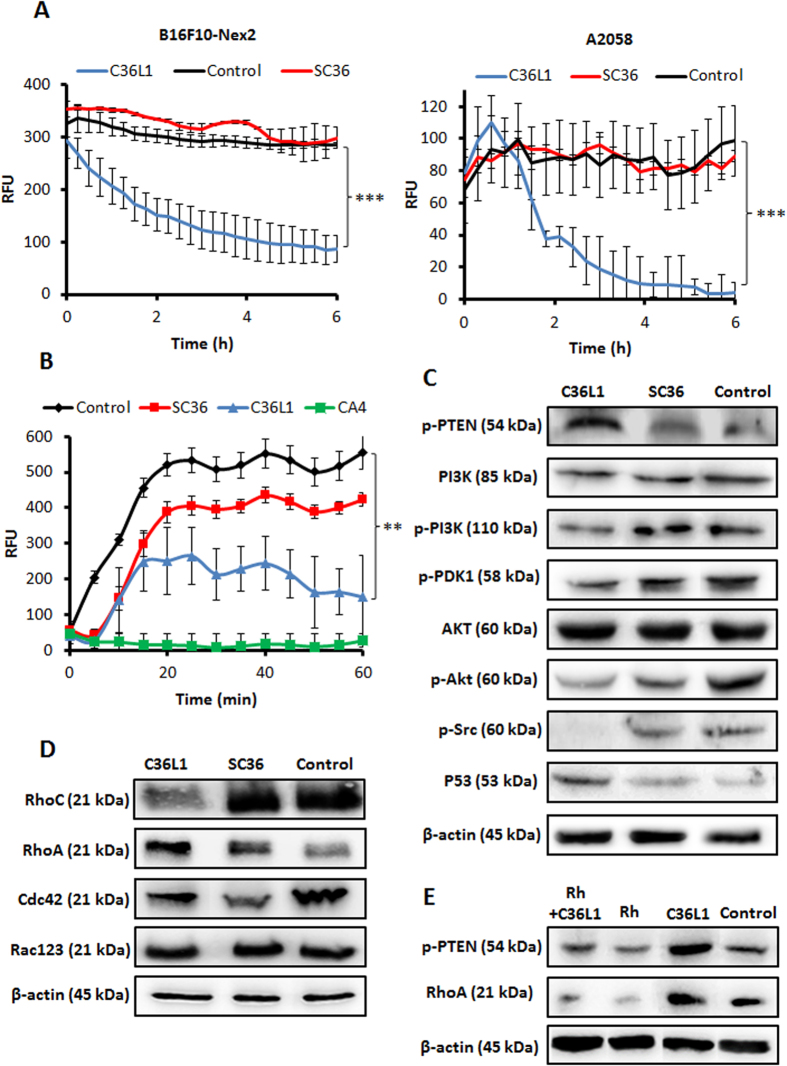
Microtubule de-stabilization and cell signaling. (**A**) B16F10-Nex2 and A2058 cells expressing baculovirus-transduced fluorescent microtubules were incubated with C36L1 and SC36 at 5 nmoles/10^3^ cells and microtubule integrity was quantified in real time as described in Methods; (**B**) C36L1 and SC36 (15 nmoles/well) were incubated with tubulin and polymerization kinetics was measured. CA4 (7.5 nmoles/well) were used as positive control (**p < 0.01; **p < 0.001, in relation to untreated control); (**C**) Lysates from B16F10-Nex2 cells, previously treated with peptides at 0.25 nmole/10^3^ cells for 18 h at 37 °C, were analyzed by Western blotting. The following antibodies to p-PTEN (S380), total PI3K, p-PI3K (p110α), p-PDK1 (S241), total Akt, p-Akt (S473), p-Src (T416) and total p53, were used; (**D**) The Rho-GTPase family proteins were also evaluated with antibodies to RhoC, RhoA, Cdc42 and total Rac123; (**E**) Lysates from B16F10-Nex2 cells, previously treated with Rh 50 μM for 12 h and then incubated with C36L1 at 0.4 nmol/10^3^ cells for 18 h at 37 °C, were analyzed by Western blotting for p-PTEN (S380) and RhoA levels. β-actin was used as total protein loading control.

Microtubule targeted antimitotic drugs are classified into two main groups: the microtubule-de-stabilizing agents, which inhibit microtubule polymerization and the microtubule-stabilizing agents, which stimulate microtubule polymerization^24^. To investigate the effect of C36L1, the microtubule cytoskeleton was monitored by live-cell imaging using B16F10-Nex2 cells transduced with a modified insect virus (baculovirus) containing a tubulin-red fluorescent fusion protein construct (CellLight^®^, Life Technologies). The fluorescence of live murine and human melanoma cells was monitored and quantified for 6 h during treatment with 5 nmoles/10^3^ cells of C36L1, SC36 and negative control, and we observed that C36L1 drastically reduced microtubule fluorescence rate compared to negative controls (Fig. 6a), indicating that the microtubule network was de-stabilized during the incubation period. Videos showing the kinetics of microtubule depolymerization in tumor cells during the incubation with C36L1, compared with the negative control (RPMI medium) or DMSO 5% (a negative control of depolymerizing cell death) are available, respectively (Supplementary Video S4 and S5). In addition, C36L1 induced a significant delay in recombinant tubulin polymerization and promoted depolymerization after 40 min (Fig. 6b), using a fluorescent recombinant tubulin polymerization assay kit (Cytoskeleton, Denver, CO).

### C36L1 suppresses Pi3k/Akt signaling by regulating PTEN in a Rho-GTPase dependent way

The PI3K (phosphatidylinositol 3-kinase)-AKT pathway plays a significant role in melanoma, with regulation of important cellular properties, such as proliferation, motility and invasion^25^, that have been modified with C36L1 treatment. Western blotting was carried out with protein lysates of B16F10-Nex2 cells previously incubated with C36L1 peptide at 0.25 nmole/10^3^ cells. C36L1 treatment decreased the expression of the catalytic phospho-PI3K (p110α), phospho-PDK1 (S241), phospho-AKT (S473) and increased the levels of constitutive PTEN (phospho-PTEN at S380) (Fig. 6c). In addition, phospho-Src (S416) was drastically reduced and increased levels of total p53 were seen (Fig. 6c). Finally, the levels of the small proteins of the Rho-GTPase family (well-known PTEN regulators) were investigated. RhoA increased, unlike RhoC that was reduced after treatment with C36L1 peptide (Fig. 6d). Levels of Cdc42 and Rac-123 were not affected by peptide treatment.

Previous reports have described de-stabilization of MTs as a crucial process linked to the release of the guanine-nucleotide exchange factors (GEFs), which activates Rho-GTPases, such as RhoA, for further regulation of PTEN 26–29. In order to investigate whether RhoA mediated PTEN activity is essential for C36L1's function, we used a commercial RhoA inhibitor (Rhosin) previously described to specifically inhibit RhoA activity and RhoA-mediated cellular function without affecting Cdc42 or Rac1 signaling activities^26^. Thus, B16F10-Nex2 cells were incubated with 0.4 nmole/10^3^ cells of C36L1 with or without pre-incubation with Rhosin (Rh) at 50 μM for 12 h. We observed that, under these conditions, Rh downregulated RhoA and p-PTEN levels, even in the presence of C36L1, as compared to C36L1-alone treated cells (Fig. 6E), showing an intrinsic relationship between the C36L1 and RhoA activities also involving PTEN activation. The essentiality of RhoA mediated PTEN activity for C36L1’s function should depend on the concentration balance of C36L1 and RhoA and of the latter with the phosphorylation of PTEN. Using the viability phenotype, C36L1 lost its properties in the 0.2–3 nmoles/10^3^ cells range, upon high but still partial inhibition of RhoA and consequently of p-PTEN. At 5 nmoles/10^3^ cells, C36L1 killed 50% of RhoA-inhibited cells (Supplementary Figure S3).

### Antitumor activity *in vivo* against metastatic and subcutaneous melanoma

**Figure 7:**
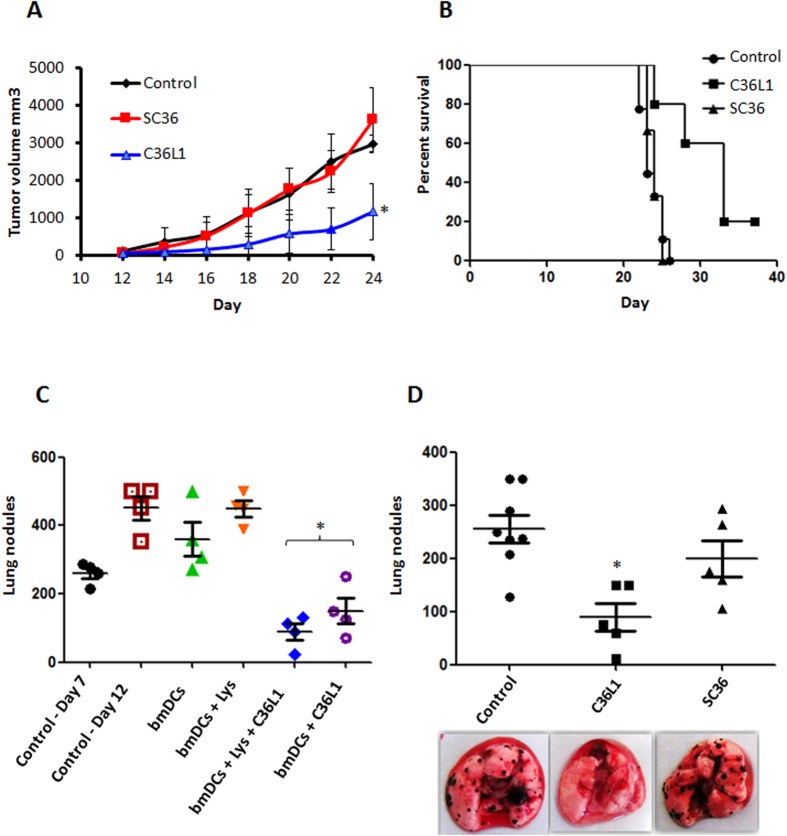
Antitumor activity of C36L1 peptide *in vivo*. (**A**) Antitumor activity of C36L1 at 10 mg/Kg peptide in subcutaneously grafted melanoma treated with peritumor injections of peptide. The tumor volume was documented during the treatment period (*p < 0.05, in relation to untreated control); (**B**) Survival kinetics of B16F10-Nex2 s.c. challenged animals, treated with C36L1 via peritumor route; (**C**) Therapeutic activity of bone marrow derived dendritic cells (bmDCs) stimulated with C36L1 and primed or unprimed with tumor cells lysate, transferred to 7 day-melanoma bearing mice. Arrest and even regression of metastatic nodules are shown (*p < 0.05, in relation to day-7 control); (**D**) Anti metastatic activity of C36L1 peptide in syngeneic murine melanoma model. Lungs were harvested for quantification of pulmonary metastatic nodules after peptide treatment. Representative images of the lungs are shown (*p < 0.05, in relation to untreated control).

Previously, we showed that C36L1 peptide displayed antitumor activity *in vivo* in a metastatic murine melanoma model^15^. Here, we show that C36L1 can also significantly reduce tumor progression of a subcutaneously grafted murine melanoma (Fig. 7a) using peritumoral administration of the peptide, and significantly prolonged mice survival. The SC36 peptide was inactive both in the subcutaneous and metastatic models of tumor growth (Fig. 7a–c). In the control group, SC36 and C36L1 groups of Fig. 7b, no animal died as a result of the experimental conditions. All animals died by humane intervention after tumor volumes have reached close to 3,000 mm^3^.

### *In vivo* antitumor activity of C36L1 depends on the immune system

The antitumor activity of C36L1 could not be reproduced in NOD/Scid/IL-2rγ^null^ immunodeficient mice (data not shown), as with two other CDR peptides with antitumor activity previously described^1^,^16^. Presently, a therapeutic protocol was used in which bone marrow dendritic cells, previously incubated *ex vivo* with C36L1, primed or unprimed with a melanoma cell lysate, and adoptively transferred to C57Bl/6 mice with growing lung metastases of B16F10-Nex2 cells, significantly protected the animals. C36L1-stimulated DCs decreased the number of metastatic nodules (Fig. 7c) exerting a therapeutic effect similar to that of the isolated peptide inoculated intraperitoneally in animals challenged endovenously with melanoma cells (Fig. 7d).

## Discussion

Previously, we demonstrated that the Ig-CDR peptide C36L1 is cytotoxic to B16F10-Nex2 melanoma cells and a panel of human tumor cells, but not against non-tumorigenic cells, such as murine melanocytes and fibroblasts, with *in vitro* IC50 values on the same concentration range^15^, suggesting the involvement of a conserved target on tumor cells. Here, we investigated the mechanisms of action of C36L1 in dose-dependent apoptotic and non-apoptotic conditions. Antitumor effects of peptide were investigated at moles/10^3^ cells. In some experiments the mole/cell concentrations varied for optimal results, but they always conserved a gap between each type-response (apoptosis at high concentrations and antitumor effects at low concentrations. Low concentrations range from 0.2 to 2 nmoles/10^3^ cells, and high concentrations range from 3 to 7.5 nmoles/10^3^ cells.

Recently, we have described a CDR peptide that bound to and altered the dynamics of actin inducing apoptosis^12^. In the present work we show that C36L1 interacts with microtubules (MTs), in agreement with its broad antitumor activity in different cancer cells. Microtubule-targeting agents are a very promising class of cancer drugs with therapeutic benefits in both hematopoietic and solid tumors^27^. A close relationship exists between cell death mediated by intrinsic apoptosis and the damages infringed in microtubules^28^.

C36L1 is apoptotic in melanoma cells through the intrinsic pathway, as evidenced by inducing chromatin condensation, DNA fragmentation and translocation of phosphatidylserine. Also, C36L1 induced ER stress, which results in the release of Ca^2+^ and is associated to loss of the mitochondrial membrane potential. As observed in the supplementary video 1, tumor cells begin to die approximately at 4 hours (240 min) after incubation with the cytotoxic concentration 5 nmoles/10^3^ cells. Before this period, almost all cells are alive, in accordance with a time dependent cytotoxic assay performed at this concentration (supplementary Figure 1). Also, before 4 hours of tumor cells incubation with C36L1 at 5 nmoles/10^3^ it is possible to observe a significant loss of mitochondrial membrane potential starting at 160 min (2.6 hours) for both B16F10-Nex2 and A2058 melanoma cells. The release of Ca^2+^ from the ER starts even earlier, at approximately 90 min (1.5 hours), demonstrating a cause and consequence relationship between these two phenomena, which further results in tumor cell death. Given the intrinsic mechanism of cytotoxicity induced by C36L1, about 60% of tumor cells lost their viability at 6 h of incubation, and there were only 10% of remaining viable cells after 18 h of treatment.

Depletion of Bcl-2 and Bcl-xl proteins, increase of caspases 3 and 9 activities, and cleavage of PARP caused by C36L1 are all well characterized apoptosis related events^29^. The peptide arrests the cell cycle at G2/M compatible with the effects on MTs. In comparison, combretastatin-A4 (CA4), which was utilized as positive control in specific experiments of this work, depolymerizes MTs, induces cell cycle arrest in the G2/M phase and mitotic catastrophe characterized by the translocation of pro-apoptotic protein Bim to mitochondria with activation of caspases-3 and caspase-9^30^.

The C36L1 peptide binds to tubulin and promotes microtubule depolymerization that led to physical and functional coupling of a condensed endoplasmic reticulum to mitochondria. This was evidenced by transmission electron microscopy. Interactions between the ER and mitochondria are both physical and functional and Ca^2+^ is a mediator of these interactions^31^. In particular, ER stress and activation of the intrinsic pathway of apoptosis are calcium-dependent processes^32^. A major determinant of the ER–mitochondria interface is the distance between organelle surfaces, which is determined by organelle movement along the cytoskeleton^33^. Regions of close proximity between mitochondria and the ER are necessary for Ca^2+^ entry into the mitochondrial matrix^34^. A recent study shows that MTs are important in the interaction between the ER and mitochondria coupling process, and that depolymerization of MTs has been associated with increased coupling in radial and central regions of the cell^31^. The C36L1 peptide may induce apoptosis by MT-dependent coupling between ER and mitochondria, inducing ER stress and Ca^2+^ dependent crosstalk between these organelles culminating in loss of mitochondrial potential and subsequent cell death by the intrinsic apoptosis pathway.

At low concentrations, i.e. when the availability of the peptide per cell is at least 10 times less than the required concentration per cell to induce apoptosis, we found that C36L1 affects other phenotypes of the cancer cell such as migration, invasion and proliferation, which are needed in the metastatic process^35^. Microtubules are essential for migration, invasion, adhesion and proliferation of melanoma^36^. The study of cellular signaling induced by C36L1 at low concentrations in B16F10-Nex2 cells showed that the peptide effectively interferes in the PI3K/Akt axis, one of the most important signaling pathways in cancer^25^. The activity of PI3K by its catalytic p110 subunit is required for the generation of PIP3, which in turn recruits and phosphorylates PDK to completely activate AKT by phosphorylation^25^,^37^. Since the active form of AKT (phosphorylation at S473) is lowered in C36L1 treatment together with the upstream regulators PDK1 and PI3K, we investigated the levels of a potential regulator of this signaling pathway, the phosphatase and tensin homolog (PTEN) protein. PTEN is responsible for dephosphorylation of PIP3 to produce PIP2, thus interfering with the activation of AKT^37^. The phosphorylated form of PTEN on serine-380 is a constitutive stable form that can be activated by dephosphorylation, thus self-maintaining a constant activation loop^38^. PTEN also regulates the activity of Src, which was described to induce the migration of glial cells by activating PI3K/AKT axis^39^. In addition, regulation of PTEN transcription is dependent of p53, the activity and levels of which have been associated to depolymerization of microtubules^40^,^41^,^42^.

The constitutive increase of active PTEN can be explained by the de-stabilization of MTs during C36L1 treatment, a crucial process that is linked to the release of specific factors, such as the guanine-nucleotide exchange factors (GEFs), which activate Rho-GTPases for further regulation of PTEN^43^,^44^,^45^,^46^. GEFs are initially associated with MTs and are released to cytosol during MTs depolymerization to activate Rho-GTPases, such as RhoA in response to GEF-H1^43^,^44^,^47^. Within the GTPases family, the Rho subclass includes RhoA, the most studied member, and two closely related homologs, RhoB and RhoC^48^. Once activated, RhoA induce Rho-associated kinase (ROCK) activation, which upregulates the activity of PTEN^49^. Specific GEFs may be released during treatment with C36L1 by depolymerization of MTs, and may result in the activation of RhoA.

Thus, we suggest the involvement of RhoA in the activation of PTEN given the fact that higher level of this GTPase was found in B16F10-Nex2 cells after C36L1 treatment compared to SC36 and a negative control. Moreover, pPTEN was downregulated by RhoA inhibitor Rhosin, even in the presence of C36L1, suggesting that this specific PTEN activation pathway is maintained during C36L1 induced RhoA activity. In addition, using the viability response phenotype of C36L1, we observed that downregulation of RhoA and consequently of PTEN in tumor cells resulted in loss of C36L1 properties at low concentrations. At high concentrations (5 nmoles/10^3^) C36L1 killed only 50% of RhoA-inhibited cells, demonstrating that PTEN is also necessary for induction of apoptotic stimuli. In fact, previous works have described that apoptosis induced by different agents can be mediated by PTEN in malignant melanoma cells, mainly through the upregulation of proapoptotic pathways which involve caspases and the downregulation of antiapoptotic proteins such as Bcl2, also observed by C36L1 treatment^50^.

In contrast to RhoA, RhoC expression, which is downregulated by C36L1, is associated with highly metastatic tumors^51^,^52^, in a PI3K/Akt dependent way^53^. Furthermore, previous studies have shown that downregulation of a specific member of the Rho-GTPases family is associated with a proportional increase of another member, in this case Rho-A, whose stability in the cytosol is favored by the increasing availability of GDIs (guanine nucleotide dissociation inhibitor), which inhibits cytosolic degradation of Rho-GTPases^54^.

Most importantly, C36L1 exerted antitumor activity *in vivo*, probably by exerting a direct cytotoxic effect on subcutaneously grafted melanoma, when administered via a peritumoral route. In a more systemic model, however, protection by this peptide injected intraperitoneally in syngeneic mice depended on the immune system. The possible involvement of dendritic cells mediating anti-metastatic responses as with other CDR peptides in a prophylactic protocol to treat melanoma^16^, was also examined with C36L1. Dendritic cells (DCs) have been widely studied to induce tumor-specific immunity^55^, and microtubule depolymerization agents have been described to induce maturation of DCs^56^, enhancing antigen presentation and antitumor immunity in DC-based cancer vaccines^57^,^58^,^59^,^60^. Using a therapeutic protocol, in which animals were challenged with B16F10-Nex2 melanoma cells 7 days before administration of syngeneic bone marrow DCs stimulated with C36L1 and primed or unprimed with tumor lysate, a remarkable protective effect of the peptide was obtained. Since C36L1 is unrelated with melanoma antigens, it is clear that it played an immunomodulatory role, which presently is being further investigated. We conclude that C36L1 peptide displays an important role in the protection against malignant melanoma *in vitro* and *in vivo*, being a novel candidate for the class of drugs that acts on the microtubule network and those that exert immunomodulatory effects on DCs.

## Methods

### Ethics statement

This work is part of project 2010/51423-0 granted by the State of São Paulo Research Support Foundation (FAPESP), Brazil, following the appropriate scientific and ethical considerations. Animal experiments were carried out using protocols in accordance with the Ethics Committee of Federal University of São Paulo, Brazil, CEP 1234/2011. In the subcutaneously grafted tumor experiment, no animals died as a result of the experimental conditions before day 24. All animals died by humane intervention when tumor volumes reached up to 3000 mm^3^ or at the end of the experiment on day 24. The standard clinical symptoms adopted in the lab, that indicate deteriorating health conditions requiring euthanasia before the end of the experiment are weight loss, inappetence, weakness (inability to feed and drink), infection (with systemic signs of illness) or any severe sign of organ dysfunction. All experimental protocols were approved by Ethics Committee of Federal University of São Paulo, Brazil, CEP 1234/2011, and the specific Project presented by the Experimental Oncology Unit (UNONEX).

### Mice, metastatic and subcutaneous melanoma model

All experiments were performed in accordance with relevant guidelines and regulations of the Ethics Committee for Animal Experimentation of Federal University of São Paulo. C57BL/6 6-8 weeks old mice were obtained from CEDEME (Centro de Desenvolvimento de Modelos Experimentais) from the Federal University of São Paulo (UNIFESP), free from contamination or any type of pathology and were housed in ventilated racks (ALESCO) in specific pathogen free conditions (SPF). Animals were monitored three times per week to assess tumor burden, behavior and distress. For metastatic melanoma assay, male C57Bl/6 mice were intravenously challenged with 5 × 10^5^ syngeneic B16F10-Nex2 viable cells in 0.1 mL of RPMI medium without BFS (bovine fetal serum) for each mouse. Animals were challenged with tumor cells and treated in the next day with intraperitoneal (i.p.) doses of 300 μg (10 mg/kg) of each peptide in consecutive days, or with vehicle control (1% DMSO in PBS). Five animals were used for treatment with C36L1 and SC36 peptides and eight animals were used in the untreated control group. After 14 days, mice were euthanized by cervical dislocation and lungs were harvested and inspected for metastatic colonization. Melanotic nodules were quantified and photographed in a stereo microscope (Magnification, ×4) (Nikon, Tokyo).

For subcutaneous (s.c.) melanoma assay, male C57Bl/6 mice (five per group) were subcutaneously grafted in the right flank with 5 × 10^4^ syngeneic B16F10-Nex2 viable cells. At this tumor cell density only four animals developed tumors in each group. Animals were subjected to 10 peritumoral daily doses of 300 μg (total 10 mg/kg) of each peptide. DMSO (1%) in PBS was used as vehicle control. Treatment started after 12 days and tumor sizes were measured every other day with a caliper. Tumor volume (V) was calculated by the formula V = 0.52 × d^2^ × D, where d and D correspond to the short and long diameters of the tumor, respectively. Animals were monitored three times a week to assess tumor burden, behavior and general health status. Mice were euthanized by cervical dislocation carried out by expert individuals at the end of experiments, or before, should the tumors ulcerate, reach the maximum allowed volume of 3000 mm^3^, or in the case of severe health deteriorating conditions. As tumor volume measurements were performed in alternate days, in a few cases of fast tumor growth between the last 2 days, the tumor volume exceeded 3000 mm^3^. These animals were immediately euthanized, and the values included in the mean calculation for that point.

### Peptides

Peptides were purchased from Peptide 2.0 (Chantilly, VA). C36L1 peptide (KSSQSVFYSSNNKNYLA-NH2) and the scramble SC36 (NQSYSFSKASLNVSYKN-NH2) control peptide, were both amidated at the C terminus. The purity obtained in the synthesis was 95–98%, and was determined by High-performance liquid chromatography (HPLC) using a C18 column subsequently analyzed by mass spectrometry, according to manufacturer’s specifications. They were diluted in 1% dimethyl sulfoxide (DMSO), and then to a final solution in RPMI 1640 medium.

### Tumor cell lines and cell culture

The murine melanoma cell line B16F10-Nex2 is characterized by adherence, spindle or stellate morphology, darkly melanotic cells, fast growing *in vitro* and *in vivo* forming black tumor masses and black nodules in the lungs when injected subcutaneously and intravenously, respectively, in syngeneic H-2^b^ C57Bl/6 mice. The original B16F10 lineage was obtained from the Ludwig Institute for Cancer Research, São Paulo branch. The present B16F10-Nex2 cell line was isolated at the Experimental Oncology Unit, Federal University of São Paulo, and deposited in the Banco de Células do Rio de Janeiro (BCRJ), reg. 0342. Human melanoma cell line A2058 was provided by the Ludwig Institute for Cancer Research, São Paulo, Brazil. Both cell lines were cultured at 37 °C, under humid atmosphere and 5% CO_2_, in RPMI-1640 medium with 10 mM N-2-hydroxyethylpiperazine-N2 ethane sulfonic acid (HEPES), 24 mM sodium bicarbonate, 40 mg/L gentamicin, pH 7.2 and 10% fetal bovine serum (FBS).

### Cell viability assay

Tumor cells were seeded in 96-well plates and then incubated with the vehicle (negative control), 1% DMSO in complete RPMI medium, and different concentrations of C36L1 and SC36 peptides for 24 h. Viable cells were quantified using the MTT (3-[4,5-dimethylthiazol-2-yl]-2,5-diphenyltetrazolium bromide) (Sigma-Aldrich, St. Louis, MO) assay. After incubation with the peptides, MTT solution (5 mg/ml) in phosphate-buffered saline (PBS) was added to the cells, followed by incubation for 3 h at 37 °C. Absorbance was measured in a microplate reader at 570 nm (SpectraMax-M2, Molecular Devices Software Pro 5.4, Sunnyvale, CA). Cell viability was expressed as percent values in comparison with untreated control cells.

### Chromatin condensation

B16F10-Nex2 melanoma cells (2 × 10^4^) were seeded on round glass coverslips sinked in a 24-well plate and incubated with control vehicle and 7.5 nmoles/10^3^ cells of C36L1 and SC36 peptides for 5 h. After incubation, cells were washed with PBS, fixed in methanol for 15 min at room temperature, washed again and stained with 2 μM of Hoechst 33342 (Invitrogen) for 10 min. Slides were prepared with the cover slips and cells were visualized in an Olympus BX-51 fluorescence microscope (magnification, ×600). Images were processed with ImageJ software.

### Caspase assay

The activities of caspases 2, 3, 6, 8, and 9 were measured using the ApoTarget Caspase Sampler kit (Invitrogen), according to manufacturer's instructions. Briefly, 5 × 10^5^ B16F10-Nex2 cells were incubated with C36L1 peptide at 1.2 nmoles/10^3^ cells for 2 h at 37 °C. Total proteins were extracted in 50 μl of chilled Cell Lysis Buffer (Invitrogen) on ice for 10 min. The suspension was centrifuged at 10000 × *g* and the supernatant was collected and transferred to a fresh tube. Total protein concentration was determined using the Bradford method. Protein samples from both C36L1 and control groups, at 4 mg/ml, were incubated with 200 μM of substrates DEVD-pNA (caspase 3), VEID-pNA (caspase 6), IETD-pNA (caspase 8), and LEHD-pNA (caspase 9), at 37 °C for 2 h in a 96-well plate. Absorbance was measured at 400 nm in a microplate reader (SpectraMax-M2e, Molecular Devices).

### DNA degradation assay

B16F10-Nex2 cells (10^5^) were seeded in 6-well plates and incubated with negative control, C36L1 and SC36 peptides at 6 nmoles/10^3^ cells for 12 h. After incubation period, cells were lysed in TELT buffer (50 mM Tris–HCl pH 8.0, Triton X-100 0.4%, 2.5 mM EDTA pH 9.0, and 2.5 M LiCl) and DNA was isolated as previously described^61^. The extracted DNA was subjected to electrophoresis on 1% Agarose gel previously stained with ethidium bromide (0.5 μg/ml) in TBE buffer (2 mM EDTA, 90 mM Tris–HCl, 90 mM boric acid, pH 8.0) at 100 V. A 1-kb ladder molecular weight standard was used (Gibco, Grand Island, NY). Images were acquired using a digital camera (Kodak, EDAS DC290) under ultraviolet light (UV).

### Annexin V and propidium iodide labeling

B16F10-Nex2 cells (5 × 10^5^) were cultured in 6-well plates and further incubated with peptides C36L1 and SC36 at 1 nmole/10^3^ cells or complete medium (negative control) for 18 h at 37 °C. After incubation, cells were washed three times and processed using Annexin V-FITC Apoptosis Detection Kit (Sigma-Aldrich, St. Louis, MO). Cells were incubated for 10 min at room temperature with binding buffer (10 mM HEPES/NaOH, pH 7.5, 140 mM NaCl and 2.5 mM CaCl_2_) in presence of propidium iodide (PI) and FITC-labeled annexin V (AV) and analyzed by flow cytometry (BD Bioscience FACSCanto II equipment, Franklin Lakes, NJ), using FlowJo software (TreeStar Inc., Ashland, OR).

### Transmission electron microscopy

B16F10-Nex2 cells (5 × 10^4^) were seeded in 6-well plates. Cells were then incubated with peptide C36L1 at 1.2 nmoles/10^3^ cells for 18 h at 37 °C and processed as previously described^12^. Images were acquired in a Jeol 1200 EXII electron microscope (Tokyo, Japan).

### ER condensation assay

B16F10-Nex2 melanoma cells (2 × 10^4^) were incubated with C36L1 in four chamber dishes at 5 nmoles/10^3^ cells in different periods and further ER integrity was assessed with an ER red fluorescent probe ER-Tracker (Life-technologies^TM^) following the manufacturer instructions. After incubation, tumor cells were incubated with ER-Tracker for 15 min, the ER probe was removed and cells were washed in PBS and immediately observed in a fluorescence microscope (BioStation, Nikon).

### Mitochondrial membrane potential (Δψm) assay

B16F10-Nex2 cells (2 × 10^4^) were pre-incubated with the cationic lipophilic dye tetramethylrhodamine ethyl ester (TMRE), and then with peptide C36L1 and SC36 at 5 nmoles/10^3^ cells. The fluorescence of different cell fields was measured every 10 min for 18 h of incubation in a time-lapse BioStation fluorescence microscope under controlled humidity, temperature (37 °C) and CO_2_ (5%) (Nikon, Tokyo). Subsequently, the intensity of fluorescence was quantified with NIS-Elements Imaging software (Nikon, Tokyo).

### C36L1 effects on Ca^2+^ distribution

Release of Ca^2+^ from ER, upon treatment with C36L1 was determined using the kit for detection of cytosolic calcium levels Fluo 4NW (Invitrogen). B16F10-Nex2 (2 × 10^4^) cells were seeded in 96-well plates and pre-incubated with or without 2 μM of Thapsigargin (Enzo Life Science) for 1 h. The supernatant was then removed and 100 μL of Ca^2+^ staining solution was added (according to manufacturer's specifications) for 45 min at 37 °C. After incubation with the calcium probe, cells were treated with the peptide C36L1 at 5 nmole/10^3^ cells and fluorescence readings were obtained at 494 nm excitation and 516 nm emission every 60 seconds, for 6 h, in a plate spectrophotometer (Spectra Max m2e, Molecular Devices).

### Cell lysate extracts and Western blotting

Tumor cells (10^6^) were incubated with different concentrations of peptides C36L1, SC36 in complete RPMI 1640. Cells were then washed in PBS and lysed by adding 100 μL of SDS sample buffer (62.5 mM Tris-HCl, pH 6.8 at 25 °C, 2% w/v SDS, 10% glycerol, 50 mM DTT, 0.01% w/v bromophenol blue). Lysates were transferred to microcentrifuge tubes and heated at 95 °C for 5 min, and then, protease and phosphatase inhibitors were added. Total proteins from each sample were separated in SDS electrophoresis gel, transferred to a nitrocellulose membrane and further analyzed by Western blotting as described elsewhere (Massaoka, *et al.* 2012). Full-length blotted membranes were cut according to the molecular weight of each protein, based on the pre-stained protein standard (Novex Sharp, Life technologies). Sections were further analyzed by incubation with the following highly specific primary monoclonal antibodies: mouse anti-β-actin (for total protein loading control) and the rabbit antibodies: anti-phospho PDK1 (S241), anti-phospho Akt (S473), anti-total-Akt, anti-phospho PTEN (S380), anti-total PI3 Kinase p85, anti-PI3 Kinase p110α, anti-phospho Src (T416), anti-total p53, anti-RhoC, anti-total-Rac (1, 2 and 3), anti-RhoA, anti-Cdc42, with secondary anti-mouse/rabbit IgG conjugated with horseradish peroxidase (HRP). All antibodies were purchased from Cell Signaling Technology (Beverly, MA). Immunoreactivity was detected using the Immobilon solution (Millipore, Billerica, MA).

### Proliferation assay

A thousand B16F10-Nex2 cells were incubated with C36L1 at low concentration 0.5 nmole/10^3^ cells in 96 well plates for 72 hours at 37 °C and 5% CO_2_. Cells in each well were quantified by the MTT colorimetric method and alternatively by cell counting using the Trypan blue dye.

### Cell cycle assay

B16F10-Nex2 cells (5 × 10^5^) were seeded in 6-well plate and incubated with peptides C36L1 and SC36 for 18 h at the low concentration 0.5 nmole/10^3^ cells. After incubation, cells were detached with PBS/Trypsin/EDTA and fixed in ethanol 70% on ice for 15 min. Cells were then incubated with PI solution (50 μg/ml PI, 0.1 mg/ml RNAse A and 0.05% Triton X-100). Tumor cell fluorescence was determined by flow cell cytometry in a Facs Canto II (BD, bioscience). Data were further analyzed with the Dean Jett Fox algorithm for cell cycle using FlowJo software (Tree Star Inc., Ashland, OR).

### Migration (wound-healing) assay

Tumor cells (3 × 10^5^) were seeded in 12-well plates and incubated with peptides C36L1 and SC36 at low concentration of 0.3 nmole/10^3^ cells for 18 h. The cell monolayer was then wounded with a 1 mL pipette-tip and the cell migration was observed in an inverted microscope (Nikon, Tokyo).

### Matrix invasion

Transwell chambers (BD Biosciences) with polycarbonate filters (12 mm diameter and 8 μm of pore) were incubated with 56 μL of Matrigel (BD Bioscience) 1:12 v/v for 30 min at 37 °C. Tumor cells (2 × 10^5^) were seeded and C36L1 and SC36 peptides 0.3 nmole/10^3^ cells were added in 500 μL of RPMI 1640 without FBS and incubated at 37 °C for 24 h. The lower chamber of the transwell contained 750 μl of RPMI with 10% BFS as apoptotic factor. Invading cells in the other side of the transwell membrane were fixed in absolute methanol for 10 min at 37 °C, stained with Giemsa for 15 min at 37 °C and washed in PBS. Transwell membranes were then excised and analyzed on glass slides with the aid of an inverted microscope (Nikon, Tokyo).

### Confocal fluorescence microscopy

B16F10-Nex2 cells (4 × 10^4^) were seeded in round coverslips and incubated with 1.5 nmoles/10^3^ cells of biotinylated C36L1 and SC36 peptides for 30 min at 37 °C. Methanol was used for cell fixation for 10 min at room temperature. Cells were permeabilized in 0.1% Triton X-100, blocked with gelatin 2% at 37 °C for 10 min, washed in PBS and incubated with a secondary solution (10 μg/ml DAPI and 1:200 streptavidin FITC in water) for 15 minutes at 37 °C. To determine colocalization with microtubule cytoskeleton, the same protocol was carried out with primary anti-alpha-tubulin antibody (Santa Cruz, Biotecnology, Inc) incubated after cell permeabilization. Secondary anti-IgG PE antibody (Sigma) was used at 1:500 v/v. Images were taken in a Carl Zeiss LSM780 confocal microscope (magnification, ×1000).

### Chemiluminescent dot-blotting

Peptide C36L1 binding to microtubule structures was determined by chemiluminescent (CL) dot-blotting carried out as described elsewhere with modifications^62^. Peptides C36L1, SC36 (3 μg) and an irrelevant CDR derived peptide (CE48-H2, INSGGGGTYYADSVKG-NH2), or vehicle (1% DMSO in milli-Q water) in 3 μL each, were applied on nitrocellulose membranes. These were blocked with 0.05% PBS-Tween and 5% of BSA (bovine serum albumin) in milli-Q water for 1 h at room temperature. B16F10-Nex2 cell protein lysate were prepared with non-denaturating protein extraction buffer, following the manufacturer’s instructions (Cell Signaling, Beverly, MA). Protein was quantified using Bradford’s method (Biorad). Cell lysates (20 μg/ml) were applied on nitrocellulose membranes and incubated over-night at 4 °C. A negative control (peptide coating solution) was run with cell lysate vehicle (PBS-Tween 0.05%) alone. After incubation, membranes were washed three times in 0.05% PBS-Tween for 10 min, and further incubated with anti-alpha tubulin antibody (Santa Cruz Biotechnology, Inc) for 1h at 37 °C. After three washes in 0.05% PBS-Tween membranes were incubated with anti-rabbit IgG-HRP antibody for 1 h at 37 °C. Immunoreactivity was determined using the Immobilon solution (Millipore, Billerica, MA). Readings were made in Uvitec Alliance 2.7 (UK, Cambridge). Different experimental and control dot-blots were set as follows: C36L1/MT (Tumor cell lysate and membranes-immobilized C36L1 peptide, revealed with anti α-tubulin antibody); SC36/MT (the same with SC36 peptide); CE48-H2/MT (the same with CE48-H2 inactive CDR peptide control); Vehicle/MT (Milli-Q water, tumor cell lysate and α-tubulin antibody). No interaction between peptides and the antibody was observed.

### Tubulin polymerization assay

The Microtubule Polymerization/Depolymerization fluorescence kit (Cytoskeleton, Denver, CO) was used. Black bottom 96-well plate was warmed to 37 °C. Peptide C36L1 or SC36 was added (15 nmoles) to 50 μL of the reaction solution (Buffer-1, 80 mM (PIPES) piperazine-N-N’-bis [2- ethane sulfonic acid] sodium salt; 2.0 mM MgCl; 0.5 mM EGTA (bis ethylene glycol N,N,N’,N’- tetra acetic acid), pH 6.9, with 10 μM fluorescent reporter (Cytoskeleton, Denver, CO), added to 150 μL tubulin glycerol buffer (80 mM PIPES sodium salt; 2.0 mM MgCl; 0.5 mM EGTA, pH 6.9, 60% v/v glycerol), 4.4 μL GTP stock solution at 100 mM, and 85 μL of 10 mg/ml tubulin stock solution, in each well. Readings were done every 5 min in a fluorescence microplate reader (SpectraMax-M2e, Molecular Devices Software Pro 5.4, Sunnyvale, CA). Combretastatin A4 (CA4) at 7.5 nmoles/50 μL was used as a control of tubulin polymerization inhibition.

### Live-cell imaging of microtubule dynamics

Peptide interaction with the microtubules was investigated in real time on viable melanoma cells. A cytotoxic assay was performed with B16F10-Nex2 cells previously modified by viral transduction for the expression of red fluorescent tubulin according with the manufacturer's specifications (CellLight® Reagents −2.0 BacMam, Life Technologies). After transduction, 2 × 10^4^ cells were incubated with C36L1 and SC36 at 5 nmoles/10^3^ cells and fluorescent images were taken at 5 min intervals during 8 h using the time-lapse BioStation fluorescence microscope (Nikon Instruments, Inc, Melville, NY), under controlled humidity, temperature (37 °C) and CO_2_ (5%). Fluorescence quantification was performed with the NIS-Elements Imaging software (Nikon Instruments, Inc, Melville, NY).

### Bone marrow-derived dendritic cells (BMDCs) and adoptive transfer therapy

BMDCs were obtained from the femurs of C57Bl/6 mice after euthanasia. The femurs were flushed with RPMI-1640 medium and red0 blood cells in suspension were lysed with ACK lysing buffer (150 mM NH_4_Cl, 1.0 mM KHCO_3_, 0.1 mM EDTA) for 5 min. The remaining cells from each femur were suspended in 20 ml of RPMI-1640 containing 10% FBS, 2 mM L-glutamine, 1% of nonessential amino acids, 100 U/mL penicillin and 100 μ g/mL streptomycin) supplemented with 25 ng/mL of GM-CSF and 25 ng/mL IL-4 (BD Bioscience Franklin Lakes, NJ). The cell suspension (2 ml) was incubated in a Petri dish at 37 °C with 5% CO_2_. On day 5, 1 mL of the medium was replaced by equal volume of fresh medium with GM-CSF. Non-adherent cells were discarded and adherent cells were collected and used in subsequent tests. More than 92% cells were CD11c+ as evaluated by flow cytometry. Dendritic cells were pulsed with the lysate of a subcutaneous B16F10-Nex2 tumor in the proportion of 10:1 dendritic cells: tumor cells (lysate) and with 50 μg/ml of C36L1 peptide. After incubation with tumor cell lysate and peptide, mature dendritic cells (maDCs) were used for adoptive therapy in melanoma developing animals. C57BL/6 syngeneic mice were previously injected endovenously with 5 × 10^5^ B16F10-Nex2 cells. On the day 7 after tumor challenge, lungs had already been colonized by tumor cells and mice were treated with 5 × 10^5^ maDCs into five experimental groups (n = 4 per group): Control (vehicle), maDCs only, maDCs pulsed with tumor cell lysate (L), maDCs stimulated with C36L1 only (maDCs + C36L1) at 100 μg/ml and maDCs pulsed with tumor cell lysate and treated with C36L1 (maDCs + L + C36L1). On the day 12, mice were euthanized and lung metastatic nodules were quantified.

### Statistical Analyses

The software GraphPad Prism 5.0 (San Diego, CA) was utilized for all tests. Statistical difference between groups was compared by Student’s t-test. Differences in survival time and rate were evaluated by the Kaplan-Meier survival curves. *P* values are indicated by *p < 0.05, **p < 0.01 and ***p < 0.001. Absence of * indicates non-significant differences.

## Additional Information

**How to cite this article**: Figueiredo, C. R. *et al.* A novel microtubule de-stabilizing complementarity-determining region C36L1 peptide displays antitumor activity against melanoma *in vitro* and *in vivo*. *Sci. Rep.* **5**, 14310; doi: 10.1038/srep14310 (2015).

## Supplementary Material

Supplementary Information

Supplementary Video S1

Supplementary Video S2

Supplementary Video S3

Supplementary Video S4

Supplementary Video S5
