# Supplementary material for: A novel microtubule de-stabilizing complementarity-determining region C36L1 peptide displays antitumor activity against melanoma in vitro and in vivo
Source: Sci Rep. 2015 Sep 22;5:14310. doi: 10.1038/srep14310 (PMC4585759; doi:10.1038/srep14310)
Supplement: Supplementary Information [file srep14310-s1.doc]

**A novel microtubule de-stabilizing complementarity-determining region C36L1 peptide displays antitumor activity against melanoma *in vitro* and *in vivo***

**Carlos R. Figueiredo^1^, Alisson L. Matsuo^1^, Ricardo A. Azevedo^2^,** **Mariana H. Massaoka^1^, Natalia Girola^1^, Luciano Polonelli^4^, Luiz R. Travassos^1,3^***

^1^Department of Microbiology, Immunology and Parasitology, Federal University of São Paulo (UNIFESP), São Paulo, São Paulo, Brazil.

^2^Department of Immunology, Institute of Biomedical Sciences, University of São Paulo, São Paulo, SP, Brazil.

^3^Recepta Biopharma, São Paulo, SP, Brazil;

^4^Microbiology and Virology Unit, Department of Biomedical, Biotechnological and Translational Sciences, Universitá degli Studi di Parma, Parma, Italy.

* luiztravassos@gmail.com

**Supplementary information**

**Supplementary Figures and legends**

**
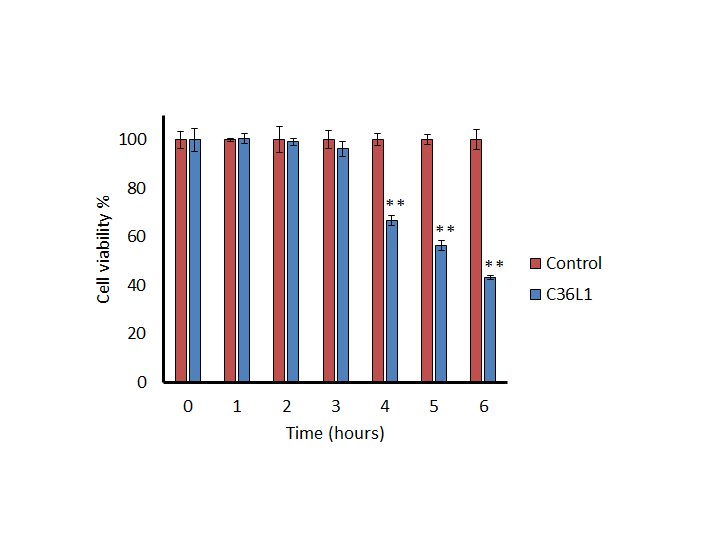
**

**Supplementary Figure S1.** Viability of B16F10-Nex2 cells at different times of incubation with C36L1 at 5 nmoles/10^3^ cells (**, p < 0.01 in relation to untreated control).

**
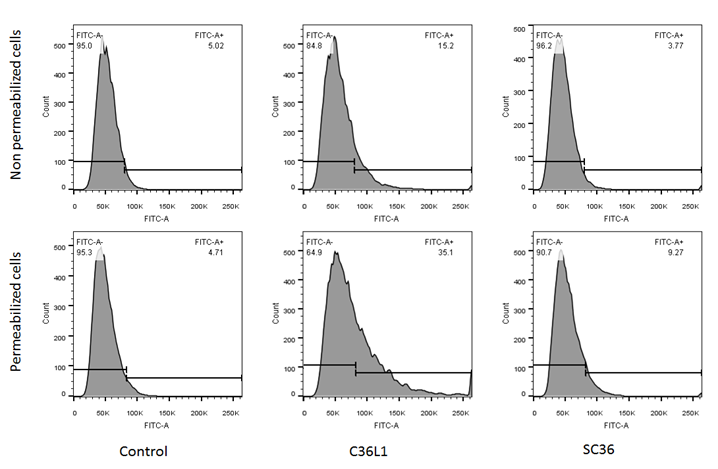
**

**Supplementary Figure S2**. Internalization of C36L1 and SC36 in B16F10-Nex2 cells. 2x10^6^ Cells were incubated with 0.15 nmol/10^3^ cells of peptides (300 μg or 150 μM). Tumor cells were then fixed in ethanol 70% in ice and permeabilized or not with 0.1% Triton X-100 in PBS in ice. Cells were washed and incubated with streptavidin-FITC for 15 min, washed and analysed by flow cytometry (BD Bioscience FACSCanto II equipment, Franklin Lakes, NJ).

**
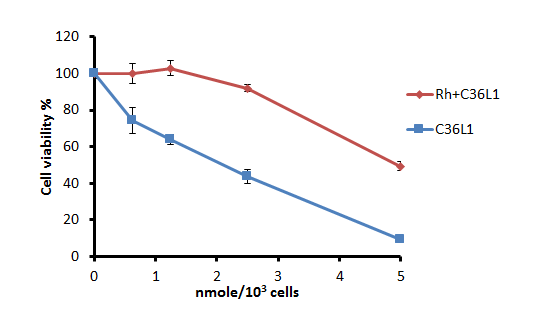
**

**Supplementary Figure S3.** Functional activity of C36L1 in B16F10-Nex2 cells previously incubated with Rhosin (Rh). (A) 1×10^4^ B16F10-Nex2 cells were previously incubated with Rh at 50 µM and then treated with different concentrations of C36L1 for 12 h (**, p < 0.01 in relation to untreated control).

**Supplementary Video legends**

**Supplementary Video S1.** Cytostatic and cytotoxic effects of C36L1 peptide monitored in real time at 37 °C and 5% CO_2_. B16F10-Nex2 cells (2 × 10^4^) were incubated with C36L1 at 5 nmoles/10^3^ cells and cell morphology was monitored by time-lapse microscopy for 24 h in a time-lapse BioStation microscope (Nikon Instruments, Inc, Melville, NY). Red arrows indicate major cytotoxic effects and green arrows indicate cytostatic activity (magnification, ×100).

**Supplementary Video S2.** C36L1 induced collapse of ∆ψm in murine melanoma cells (B16F10-Nex2) observed by time-lapse microscopy at 37 °C and 5% CO_2_ during incubation with 5 nmoles/10^3^ cells for 6h (magnification, ×100).

**Supplementary Video S3.** C36L1 induced collapse of ∆ψm in human melanoma cells (A2058) observed by Time-lapse microscopy at 37 °C and 5% CO_2_ during incubation with 5 nmoles/10^3^ cells for 6h (magnification, ×200).

**Supplementary Video S4.** C36L1 was incubated with CellLight® Tubulin-RFP transduced B16F10-Nex2 cells at 5 nmoles/10^3^ cells compared to the negative control (vehicle). Fluorescent images were taken at 5 min intervals during 8 h using a time-lapse BioStation microscope (Nikon, Tokyo) (magnification, ×400). Collected images were used to create the video file with the aid of the NIS-Elements analysis software (Nikon, Tokyo).

**Supplementary Video S5.** C36L1 was incubated with CellLight® Tubulin-RFP transduced A2058 cells at 5 nmoles/10^3^ cells compared to DMSO 5% (non-microtubule depolymerizing cell death control). Fluorescent images were taken at 5 min intervals during 8 h using a time-lapse BioStation microscope (Nikon, Tokyo) (magnification, ×400). Collected images were used to create the video file with the aid of the NIS-Elements analysis software (Nikon, Tokyo).
